# The Impact of Organizational Support on Practice Outcomes in Nurse Practitioners in Taiwan

**DOI:** 10.1097/JNR.0000000000000425

**Published:** 2021-03-19

**Authors:** Li-Hui HO, Shu-Chen CHANG, Kevin KAU, Shu-Ying SHIU, Sheng-Shiung HUANG, Ya-Jung WANG, Shiow-Luan TSAY

**Affiliations:** 1 MS, RN, NP, Department of Neurosurgery, China Medical Hospital, Taiwan, Republic of China; 2 PhD, RN, Director, Department of Nursing, Chunghwa Christian Hospital, and Adjunct Assistant Professor, College of Nursing and Health Sciences, Da-Yeh University, Taiwan, Republic of China; 3 MA, Lecturer, Academic Writing Education Center, National Taiwan University, Taiwan, Republic of China; 4 MS, RN, NP, Emergency Room, Department of Nursing, Chunghwa Christian Hospital, Taiwan, Republic of China; 5 PhD, Assistant Professor, College of Nursing and Health Sciences, Da-Yeh University, Taiwan, Republic of China; 6 PhD, RN, Assistant Professor, Department of Nursing, Da-Yeh University, Taiwan, Republic of China; 7 PhD, RN, NP, Professor, College of Nursing and Health Sciences, Da-Yeh University, Taiwan, Republic of China.

**Keywords:** organizational support, job satisfaction, care outcomes, intent to leave, nurse practitioners

## Abstract

**Background:**

Nurse practitioners (NPs) in Taiwan have practiced mainly in acute care hospitals since 2006. Although organizational support and level of support have been associated with the successful integration of NP roles and effective practice outcomes, organizational support in the context of NPs in inpatient settings is an area that has been rarely explored in the literature.

**Purpose:**

The purpose of this study was to investigate the relationship between organizational support and the practice outcomes of job satisfaction, care effectiveness, and intention to leave in NPs.

**Methods:**

A national survey of 512 NPs was conducted that included a demographic characteristics datasheet, the Nurse Practitioner Primary Care Organizational Climate Questionnaire, the Misener Nurse Practitioner Job Satisfaction Scale, and the Nurse Practitioner Care Effectiveness Scale. Multiple regression analysis was applied to explore the specific factors associated with job satisfaction. The statistical significance level was set at .05 with a two-tailed test. All statistical analyses were conducted using SPSS Statistics Version 22.0 software.

**Results:**

More than half of the participants were found to be dissatisfied with their hospital managers (54.8%) and with each dimension of organizational support. Overall, 82.1% of the participants were satisfied with their current practice. A multiple regression analysis showed that the participants who perceived higher levels of organizational support in the workplace (β = .53, *p* < .001), expressed satisfaction with working with their managers (β = .25, *p* < .001), or perceived better care outcomes (β = .10, *p* < .001) reported higher job satisfaction. In addition, the participants who expressed intention to leave within 1 year (β = −.09, *p* < .001) and those with higher patient loads (β = −.09, *p* < .001) reported lower job satisfaction. Organizational support was found to explain 50% of the variance in job satisfaction.

**Conclusions/Implications for Practice:**

The results of this study highlight organizational support as the most important factor affecting job satisfaction in NPs. Therefore, administrators work to promote organizational support and improve the work environment to enhance the job satisfaction, increase the clinical practice retention, and improve the care outcomes of NPs.

## Introduction

The vast majority (99.8%) of the population in Taiwan is covered under the public National Health Insurance program (Ministry of Health and Welfare, Taiwan, ROC, 2019), which makes access to healthcare easy and affordable for covered individuals. Various factors in recent years, including healthcare system reform, a rapidly aging population, increasing acuity in hospitalized patients, restrictions on working hours for residents and physicians, and a consistently high public demand for quality care, have led to a rising demand for healthcare services. Thus, the burden on the healthcare system and the quality of care in acute care hospitals have become important concerns. To address growing healthcare demands and the shortfall in healthcare provider capabilities, the government of Taiwan introduced a formal nurse practitioner (NP) program that has been delivering healthcare using physician-nurse practitioner team-based care models for inpatients since 2006 (Chiu et al., 2016; Tsay et al., 2019). Most of Taiwan's 8,849 licensed NPs (as of May 2020) work in acute care hospitals (Ministry of Health and Welfare, Taiwan, ROC, 2020).

Although the government has mandated regulations for the education, licensing, and practice of NPs (Tsay et al., 2019), the healthcare organizations that employ NPs significantly influence the actual role played by NPs in patient care. Specific roles for NPs are often defined by the employing organization. Moreover, organizational support has been related to factors including the successful integration of the NP role, NP job satisfaction, intention to continue working as an NP, and care outcomes (Poghosyan et al., 2020; Poghosyan, Nannini, & Clarke, 2013). Although NPs are increasingly relied upon in hospitals to provide healthcare, systematic review studies have shown that NPs are capable of delivering high-quality, cost-effective care to patients in primary-care office, inpatient, outpatient surgical, and other practice settings as well (Stanik-Hutt et al., 2013). However, studies have shown that the practice of NPs is often not supported within their organizations (Poghosyan et al., 2020; Poghosyan, Nannini, & Clarke, 2013). In addition, each acute care hospital in Taiwan has its own administrative system. Thus, NPs are evaluated by managers who may be physicians, non-NPs, or NP supervisors who are unfamiliar with NP roles and functions and, therefore, may not provide to NPs adequate organizational support, resources, and rewards. The impact of organizational support on the practice outcomes of NPs in acute care settings is an issue that has received little research attention.

Organizational climate/support is one of the important factors associated with outcomes for healthcare professionals (Aiken et al., 2011). The findings of Albashayreh et al. (2019) indicate that a good work environment is positively correlated with job satisfaction. In a cross-national study, better work environments were shown to reduce the likelihood of job dissatisfaction (Aiken et al., 2011). Poghosyan et al. (2017) reported similar results, with NPs with higher scores for organizational climate showing higher levels of satisfaction with their jobs (*OR* = 1.24, 95% CI [1.12, 1.39]). In addition, a descriptive, correlational study showed that the relationship between NPs and staff was also correlated with job satisfaction (LaMarche & Tullai-McGuinness, 2009). Another large, cross-sectional online survey conducted by Halcomb and Ashley (2017) found collegiality to be the most satisfying professional aspect of work (Halcomb & Ashley, 2017).

Practice outcomes often include job satisfaction, intent to leave, and care quality (Poghosyan et al., 2020). Job satisfaction is defined as the perceived satisfaction of NPs with their clinical practice in several dimensions, including intrapractice partnership/collegiality; challenge/autonomy; professional, social, and community interaction; professional growth; time; and benefits (Misener & Cox, 2001). Moreover, job satisfaction is integral to retention efforts (Albashayreh et al., 2019), and several studies have found a correlation between job satisfaction and intention to change jobs (Applebaum et al., 2010; Masum et al., 2016; Poghosyan et al., 2017). For instance, Applebaum et al. showed that job satisfaction is inversely linked with intention to quit (Applebaum et al., 2010). The results of a study by Masum et al. (2016) also confirmed an association between higher job satisfaction and lower turnover (*r* = −.57, *p* < .01; Masum et al., 2016). Furthermore, Poghosyan et al. (2017) found that the odds of job satisfaction factors increased by approximately 20%, whereas the odds of turnover intention decreased by approximately 20%. Apart from this, the practice of NPs has been found to encompass a variety of dimensions, including providing direct patient care services, patient counseling, and health promotion education and performing administrative responsibilities, which, added together, generate heavy workloads. The results of Miller et al.'s (2005) study indicate that maintaining reasonable working hours enhances job satisfaction in NPs. One study conducted using both quantitative and qualitative methods indicates that both working hours and the duration of time worked in one's current job are statistically and significantly related to job satisfaction in NPs (Steinke et al., 2018). Patient workload is positively associated with work stress in NPs and thus negatively associated with job satisfaction (Ramoo et al., 2013). Furthermore, increased satisfaction with one's work as an NP has been shown to improve not only retention but also patient outcomes (Horner, 2017). Watts (2019) noted that work satisfaction affects patient safety and care. One review study also found that NPs experience dissatisfaction when a patient outcome is not in line with the expected outcome (Jaeger et al., 2016).

In summary, NP job satisfaction is affected by specific factors, such as organizational support, the relationship between NPs and staff, turnover intention, workload, and care outcomes. However, the studies on this issue in the literature are limited by their use of small sample sizes, specific geographic areas, and convenience sampling methodology (J.-Y. Hu et al., 2018). Little is known about which factors affect job satisfaction. Furthermore, the association between these factors and job satisfaction among NPs requires further examination using more representative samples, for example, NPs in Taiwan. Therefore, this study was designed to investigate the relationship between organizational support and the practice outcomes of job satisfaction, care effectiveness, and intention to leave using a national sample of nursing practitioners.

Organizational support theory was adopted as the framework for this study. According to this theory, the beliefs of employees relate to the extent to which the employer values their contributions and cares about their well-being. Organizational support is also valued as assurance that resources will be available when they are needed to carry out one's job effectively (Eisenberger et al., 1986). Thus, organizational support is expected to have different effects on different employee outcomes such as job satisfaction (Rhoades & Eisenberger, 2002), healthcare outcomes (Aiken et al., 2011), and turnover intention (Arshadi, 2011; Poghosyan et al., 2020). For NPs, organizational support is essential to promoting NP performance and outcomes, including improving job satisfaction and quality of care as well as reducing turnover (Poghosyan et al., 2020). In light of these concerns, the organizational support theory offers a good framework for pursuing the aims of this study.

## Methods

### Design and Participants

The researchers conducted a nationwide survey of NPs using a cross-sectional design. The participants were recruited from the membership of the Taiwan Association of Nurse Practitioners, which includes approximately 90% of all licensed NPs in Taiwan. The data were collected between October 2016 and November 2016. The inclusion criteria included holding active membership in the Taiwan Association of Nurse Practitioners, holding national NP certification, and being employed in a hospital or practice as an NP for at least 1 year. The eligible NPs were invited to participate via email. Those NPs who agreed to participate and provided informed consent were then asked to complete an anonymous survey online. The survey consisted of a demographic characteristics questionnaire, a work-related information datasheet, the Nurse Practitioner Primary Care Organizational Climate Questionnaire (NP-PCOCQ), the Misener Nurse Practitioner Job Satisfaction Scale (MNPJSS), and the Nurse Practitioner Practice Outcome Scale. Three thousand NPs were invited to participate in this study, and 512 NPs completed the survey, yielding a response rate of 17.1%. Approval for this study was granted by the institutional review board of China Medical University and Hospital Research Ethics Committee (approval number: 13MMHIS231).

### Measures

#### Organizational support

In this study, practice environment was assessed using the NP-PCOCQ, which is an NP-specific tool that was designed to measure organizational support in primary and acute care settings. The NP-PCOCQ has been shown to have satisfactory internal consistency (Cronbach's alpha = .87–.95) and construct validity (Poghosyan, Nannini, Finkelstein et al., 2013).

The NP-PCOCQ consists of 29 items in the four dimensions of (a) NP–physician relations (measuring the relationship between NPs and physicians), (b) NP–administration relations (measuring the collaboration between NPs and managers), (c) independent practice and support (measuring the support or resources for NPs in practice), and (d) professional visibility (measuring the visibility of NPs in practice; Poghosyan et al., 2019). Each item is rated on a 5-point scale ranging from *strongly disagree* (0) to *strongly agree* (4). Individual dimension scores were calculated as the sum of the items within the dimension. The total score was calculated by summing all 29 items. Higher scores indicated higher levels of the associated characteristic in the practice and, thus, a better practice environment in terms of the associated dimension. The Chinese version of the NP-PCOCQ has also shown good reliability (Cronbach's alpha = .87–.95) and construct validity, with a Cronbach's alpha value of .90 in this study.

Practice outcomes examined in this study included job satisfaction, care effectiveness, and intention to leave one's current job.

#### Job satisfaction

NP job satisfaction was measured in this study using the MNPJSS, which encompasses 44 self-administered items measured on a 6-point scale ranging from (1) *very dissatisfied* to (6) *very satisfied*. This scale includes the six dimensions of intrapractice partnership/collegiality; challenge/autonomy; professional, social, and community interaction; professional growth; time; and benefits. Dimension scores are calculated by summing all the items within a dimension. The MNPJSS total score is calculated by summing all of the items, with higher scores associated with greater job satisfaction. The Cronbach's alpha value for the entire scale was .96, with subscale alphas ranging from .79 to .94 (Misener & Cox, 2001). The Chinese version of the MNPJSS also has good reliability (entire scale: .97; subscales: .80–.93) and construct validity (C.-Y. Hu et al., 2014). The Cronbach's alpha for this study was .92.

#### Care effectiveness

NP care effectiveness was evaluated in this study using the Nurse Practitioner Care Effectiveness Scale (NPCES), which was developed by coauthor Dr. S. L. Tsay using a literature review and qualitative interviews with a large number of NPs. This scale consists of 18 items scored on a 5-point scale ranging from (1) *strongly disagree* to (5) *strongly agree* and includes the two dimensions of efficacy of care (measuring the efficacy of care in practice) and cost of care (measuring the cost of care in practice). Dimension scores represent the sum of all items in the associated dimension. The NPCES total score is calculated by summing all 18 items, with higher scores associated with better care effectiveness in NP practice. The results of the exploratory factor analysis in this study indicate that the two dimension factors explained 74.14% of the total variance and that each item had a factor loading of over .45. Thus, the NPCES is a reliable and valid scale that may be used to evaluate care outcomes in NPs. The Cronbach's value for this scale was .94 in this study.

#### Intent to leave

Intent to leave one's current job was measured using a dichotomous item asking the NPs whether they intended to leave their positions within 1 year (yes/no). This measure has been used with NPs in previous research (Poghosyan et al., 2020).

### Data Analysis

Demographic characteristics and NP practice-related information are presented in this study as percentages, means, and standard deviations (*SD*s). The correlations between the variables were examined using multiple methods. Dichotomous variables and continuous variables were examined using point-biserial correlation. The correlations between continuous variables were examined using Pearson product–moment correlation. The correlations between ordinal variables were examined using Spearman's rank correlation. The correlations between categorical variables and continuous variables were examined using the contingency coefficient.

Stepwise linear regression analysis was used to explore the factors affecting job satisfaction. Only the significant variables in the correlation analysis were entered into the stepwise linear regression model. The standardized regression coefficients (β) and adjusted *R*^2^ were also estimated by the regression models. All of the analyses were conducted using SPSS Statistics Version 22.0 (IBM, Armonk, NY, USA). Statistical significance was determined using an alpha level of .05, presented as a *p* value of < .05 and two-sided tests.

## Results

Two thirds (66.1%) of the NPs were in the 36- to 46-year age group, most (80.3%) had over 12 years of registered nurse (RN) practice, and 53.4% reported having less than 4 years of experience as an NP (1–2 years: 26.3%, 3–4 years: 27.1%). Most worked in either medical centers (50.9%) or regional hospitals (36%). Generally, the participants reported that their practice was trusted by doctors (94.7%), RNs (92.6%), and patients (94.9%). The NP job description primarily involved direct patient care and included providing physical assessments (94.6%); discussing patient care with physicians (94.1%); taking patient histories (92.3%); prescribing medications, laboratory tests, or treatments (90.2%); performing medical procedures (87.2%); making differential diagnoses (86.6%); analyzing patient data (84.7%); providing patient education (85.8%); arranging patient transfers (79.3%); and arranging patient discharges (71.1%). The average daily care load was 11–21 patients (67.2%), and 21.4% of the NPs reported an intention to leave within 1 year (Table 1).

**Table 1:** NP Demographic and Practice-Related Variables (*N* = 513)

Variable	*n*	*%*
Age (years)		
25–35	93	18.1
36–46	339	66.1
≥ 47	81	15.8
Years of RN practice		
3–5	19	3.7
6–8	37	7.2
9–11	45	8.8
≥ 12	412	80.3
Years of NP practice		
1–2	135	26.3
3–4	139	27.1
5–6	118	23.0
7–8	121	23.6
Average number of daily patients cared for		
5–10	20	3.9
11–16	148	28.8
17–21	197	38.4
22–26	63	12.3
27–31	36	7.0
> 31	49	9.6
Hospital type		
Medical center	261	50.9
Regional hospital	188	36.6
District hospital	64	12.5
Direct patient care ^a^		
Physical assessment	494	94.6
Discussing patient care with MD	491	94.1
History taking	482	92.3
Prescribing meds, laboratory tests, treatments	471	90.2
Medical procedures	455	87.2
Making differential diagnoses	452	86.6
Analyzing patients' data	442	84.7
Patient education	448	85.8
Arranging patient transfers	414	79.3
Arranging patient discharges	371	71.1
Indirect patient care ^a^		
Medical charting	467	89.5
Attending meetings	468	89.7
Coordination patient care	448	85.8
Filling out medical forms	439	84.1
Administration duty	387	74.1
Educating others (residents, nurses, etc.)	363	70.3
Intention to leave within 1 year		
Yes	110	21.4
No	403	78.6

***Note.*** RN = registered nurse; NP = nurse practitioner; MD = medical doctor.

^a^ This item was multiple choice.

Organizational support subscale scores (range: 0–4) were as follows: NP–physician relations (mean = 2.99, *SD* = 0.35), independent practice and support (mean = 2.77, *SD* = 0.32), professional visibility (mean = 2.63, *SD* = 0.50), and NP–administration relations (mean = 2.37, *SD* = 0.51). Job satisfaction subscale scores (range: 1–6) were as follows: professional, social, and community interaction (mean = 4.33, *SD* = 0.80); intrapractice partnership/collegiality (mean = 4.16, *SD* = 0.86); challenge/autonomy (mean = 4.13, *SD* = 0.79); time (mean = 3.87, *SD* = 0.88); professional growth (mean = 3.55, *SD* = 0.99); and benefits (mean = 3.12, *SD* = 0.96). Overall, 82.1% of the NPs were satisfied with their practice. The mean item scores for care effectiveness were as follows: cost of care (mean = 4.42, *SD* = 0.93) and efficacy of care (mean = 2.71, *SD* = 0.67; Table 2).

**Table 2:** Distributions of Main Research Variables (*N* = 513)

Item	Mean	*SD*
Organizational support (range: 0–4)		
NP–physician relations	2.99	0.35
NP–administration relations	2.37	0.51
Independent practice and support	2.77	0.32
Professional visibility	2.63	0.50
Subtotal	77.68	9.01
Job satisfaction (range: 1–6)		
Intrapractice partnership/collegiality	4.16	0.86
Challenge/autonomy	4.13	0.79
Professional, social, and community interaction	4.33	0.80
Professional growth	3.55	0.99
Time	3.87	0.88
Benefits	3.12	0.96
Subtotal	164.77	34.27
Care outcome (range: 1–5)		
Efficacy of care	2.71	0.67
Cost of care	4.42	0.93
Subtotal	64.11	13.63

***Note.*** NP = nurse practitioner.

The correlations among organizational support, job satisfaction, and care effectiveness are summarized in Table 2. Organizational support was positively correlated with job satisfaction (*r* = .32, *p* < .01) and care outcomes (*r* = .35, *p* < .01) as well as with the following variables: age, years of RN practice, doctors' trust, RNs' trust, patients' trust, cooperation with doctors, and cooperation with managers (*r* = .21–.46, *p* < .01). Care outcomes showed significant relationships with the following variables: years of NP practice, doctors' trust, RNs' trust, patients' trust, cooperation with doctors, and cooperation with managers (*r* = .01–.19, *p* < .05). In addition, job satisfaction was positively correlated with the following variables: doctors' trust, RNs' trust, patients' trust, cooperation with doctors, and cooperation with managers (*r* = .17–.53, *p* < .01). Furthermore, intention to leave within 1 year was found to be negatively correlated with organizational support (*r* = −.25, *p* < .01) and job satisfaction (*r* = −.31, *p* < .01). The average number of daily patients cared for also showed a negative correlation with job satisfaction (*r* = −.14, *p* < .01; Table 3).

**Table 3:** The Correlations Among Organizational Support, Care Outcome, Job Satisfaction, and Intent to Leave (*N* = 513)

Variable	1	2	3	4	5	6	7	8	9	10	11	12	13
1. Organizational support	1.00												
2. Care outcome	.35**	1.00											
3. Job satisfaction	.32**	.32**	1.00										
4. Age	.11*	.05	.09	1.00									
5. Years of RN practice	.12**	.08	.08	.29**	1.00								
6. Years of NP practice	.07	.10*	.02	.42**	.15**	1.00							
7. Doctors' trust	.32**	.15**	.25**	.04	.16**	−.01	1.00						
8. Registered nurses' trust	.32**	.15**	.29**	.08	.19**	.07	.29***	1.00					
9. Patients' trust	.23**	.13**	.17**	.07	.15**	.02	.37***	.23***	1.00				
10. Cooperation with doctors	.21**	.19**	.21**	.18**	.10*	.02	.31***	.26***	.11*	1.00			
11. Cooperation with managers	.46**	.09**	.53**	.50**	.13**	.05	.09*	.15***	.07	.11*	1.00		
12. Patient load	−.06	−.04	−.14**	−.06	−.06	−.03	.09	.11	.13	.10	.09	1.00	
13. Apply for another job within 1 year	−.25**	−.07	−.31**	−.26**	−.12**	−.01	.21***	.18***	.05	.20***	.29***	.11	1.00

***Note.*** The correlations between dichotomous variables and continuous variables were examined using point-biserial correlations. The correlations between continuous variables were examined using Pearson product–moment correlations. The correlations between ordinal variables were examined using Spearman's rank correlations. The correlations between categorical variables and continuous variables were examined using contingency coefficients. NP = nurse practitioner; RN = registered nurse.

* *p* < .05. ** *p* < .01. *** *p* < .001.

The results of stepwise multiple linear regression show that the participants with higher levels of organizational support (β = .53, *p* < .001), satisfactory levels of cooperation with their managers (β = .25, *p* < .001), and better care effectiveness (β = .10, *p* < .001) reported higher levels of job satisfaction. In addition, those who expressed the intention to leave within 1 year (β = −.09, *p* < .001) and those who had a higher-than-average patient load (β = −.09, *p* < .001) reported significantly lower levels of job satisfaction. Fifty-eight percent of the variance in job satisfaction was explained by this model (*F* = 90.9, *p* < .01, *R*^2^ = .58, adjusted *R*^2^ = .57). Organizational support was shown to be the most important predictor of higher job satisfaction, explaining 50% of the total variance in job satisfaction (Table 4).

**Table 4:** Stepwise Multiple Linear Regression Predicting Job Satisfaction

Step	β	*t*	*R*^2^	Adjusted *R*^2^	*F*
Organizational support	.53	15.09***	.50		
Cooperation with managers	.25	7.55***	.05		
Care outcome	.10	3.19***	.01		
Intention to leave within 1 year	−.09	−3.06***	.01		
Daily patient load	−.09	−3.01***	.01		
Model fit			.58	.57	90.9**

**p < .01. ***p < .001.

## Discussion

The results of this study support the precepts of organizational support theory, which posits a positive, reciprocal, and dynamic relationship between organizational support and practice outcomes related to job satisfaction. Good organizational support, better cooperation with managers, and favorable care effectiveness were each shown to strengthen NP practice. NPs who perceive higher levels of organizational support show higher levels of job satisfaction. In contrast, organizational support, intention to leave, and a high daily patient load were each shown to reduce job satisfaction. Thus, improving perceived organizational support is key to improving NP job satisfaction.

The findings of this study identified organizational support as the most important factor affecting job satisfaction in NPs. This result is consistent with the results of prior studies (Aiken et al., 2011; Albashayreh et al., 2019; Poghosyan et al., 2017), which also found a positive relationship between organizational environment quality and job satisfaction. This finding may be attributed to good organizational support fostering positive interactions among managers, NPs, and other colleagues. This positive interaction may be viewed as the organization's contribution to a positive reciprocal dynamic with NPs (Baran et al., 2012). Generally, NPs who perceive higher levels of organizational support tend to perform better when reciprocating received feedback and to report better job satisfaction. Furthermore, the participants in this study who reported being satisfied with the working relationship with their managers (β = .25, *p* < .001) reported higher job satisfaction. Other studies have reported similar findings with regard to the associations and relationships among managers, colleagues, and NPs. For example, Poghosyan, Nannini, and Clarke (2013) noted that the relationships among NPs, doctors, and managers are significant aspects of the work environment. NPs with favorable practice environments, which include better working relations with physicians and administrators, are more likely to be satisfied with their jobs (Poghosyan et al., 2017). In evidence, doctors have reported that NPs improve the quality of patient care, patient compliance levels, and productivity. Thus, the motivation of NPs improves their cooperation, which improves the effectiveness of provided healthcare and improves job satisfaction (Holleman et al., 2010; Running et al., 2008). Our results also support the inference that the vast majority of NPs (98.4%) are satisfied with their current level of collaboration with doctors. In addition, we also found a correlation between satisfaction with one's managers and job satisfaction, which is in line with the findings of a previous study (Poghosyan et al., 2017). Hospital administrators provide sufficient resources and support to promote the autonomy of NPs in practice, especially in acute care settings. NPs who perceive adequate support channel their reciprocation efforts toward better levels of job satisfaction. Thus, it is important to support the practice environment and to decrease the perception of NPs that their practice is restricted by managers.

However, the findings of this study indicate that fewer than half (45.2%) of the participants were satisfied with the support provided by managers, and the scores for organizational support also show an inadequately supportive working environment for NPs (scores ranged from 2.37 to 2.99), which is consistent with the results of a cross-national study (Aiken et al., 2011). These results may be explained by the fact that, although national NP practice policies have been implemented for over 10 years, hospitals and NPs continue to struggle to set an appropriate practice model for acute care settings. Although the regulation allowing “nurse practitioners to perform medical care with the supervision of physicians” was issued in 2016 to protect the rights of the NP practice, some hospitals currently require NPs to perform medical care on an unlimited number of patients, which significantly increases workload stress and reduces job satisfaction in NPs. Organizational support helps NPs cope more effectively with occupational stress, whereas managers affect the work environment both positively and negatively, directly impacting job satisfaction (Ribelin, 2003). Furthermore, favorable relations with healthcare administrators have been shown to improve NP job satisfaction (Poghosyan et al., 2017). The results of this study may provide a reference for hospital management. Managers should provide adequate NP staffing and organizational support to facilitate advanced levels of practice and NP job satisfaction.

Moreover, daily patient load was highlighted in this study as a risk factor affecting job satisfaction in NPs, echoing the findings of prior studies (Miller et al., 2005; Ramoo et al., 2013; Steinke et al., 2018). The NP–patient ratios in regional hospitals and medical centers in Taiwan are much higher than those in other countries (4.7–11.7 vs. 1–8.6, respectively; Aiken et al., 2012; Kleinpell et al., 2015; Morioka et al., 2017; National Health Insurance Administration, Ministry of Health and Welfare, Taiwan, ROC, 2020). In the context of organizational support theory, higher patient load represents poor job conditions that may decrease the organizational support perceived by NPs. Although the government of Taiwan defined in 2019 the range of nurse–patient ratios allowed for different levels of hospitals as fewer than nine in medical centers, fewer than 12 in regional hospitals, fewer than 15 in district hospitals, and fewer than 15 in psychiatric hospitals, no regulations currently address the NP–patient ratio. Therefore, regulation of the NP–patient ratio is needed in the near future to establish an appropriate NP–patient ratio. In the meantime, medical institutions should recruit more NPs to decrease the overall workload. Creating a more appropriate nurse–patient ratio should improve job satisfaction in NPs and allow additional time for NPs to enhance their skills and knowledge to provide better quality of care.

The results of this study are consistent with those of prior studies showing that better care outcomes increase job satisfaction (Horner, 2017; Jaeger et al., 2016; Watts, 2019). Job satisfaction is derived not only from income but also from the feelings engendered from work performed. A good practice outcome may be viewed as a work achievement. On the basis of the organizational support theory, NPs who perceive adequate organizational support should be better prepared to engage in teamwork and perceive more job satisfaction and achieve better quality of care in their practice (Poghosyan et al., 2020). If patient care outcomes do not align with expected outcomes, NPs will experience dissatisfaction (Jaeger et al., 2016). In addition to these factors, we found significant negative correlations between NP job satisfaction and intent to leave within 1 year, which is consistent with the findings of previous studies (Applebaum et al., 2010; Masum et al., 2016; Poghosyan et al., 2017). Rambur et al. (2003) reported a stronger association between intention to leave and job dissatisfaction than with career advancement (Rambur et al., 2003). Hence, administrators view fostering good work environments as a strategy for retaining NPs and increasing their job satisfaction (Poghosyan et al., 2017). In addition, our data showed that only 45.2% of the NPs in this study were satisfied with their managers. In other words, the relationship between managers and NPs is a major barrier in the NP practice. Thus, it is necessary to promote effective communication to decrease conflicts between NPs and managers.

This study was affected by several limitations. First, this study was performed in Taiwan. Thus, the findings of this study may not be generalizable to broader populations of NPs. Second, the results may be affected by response bias, as the data were collected using a self-report questionnaire. However, a combination of anonymous questionnaire design and computer technology was used in this study to mitigate potential response error. Third, the findings were limited with regard to causal inferences because of the cross-sectional design. Future research using a longitudinal design would clarify causal inferences. The dynamic interaction of these factors may affect NP job satisfaction continuously in long-term observations. Finally, low response rates are an inevitable issue in population-based questionnaire surveys. Unfortunately, the authors had no way of accessing the demographic data of nonresponse NPs without their consent. Hence, the results should be interpreted and inferred with caution. As one recent national survey on the practice of NPs also reported a low response rate (9.9%; Kleinpell et al., 2018), our response rate (17.1%) may be considered within an acceptable range. Larger sample sizes and strategies to improve the response rate are needed in future studies. Nonetheless, the results of this study provide national-based information from Taiwan. We hope that these findings will prove helpful in addressing factors affecting NP job satisfaction and will be used as a reference for management, policymaking, and efforts to improve the work environment for NPs in medical organizations.

### Conclusions

In conclusion, this study highlighted the potential factors affecting job satisfaction and practice outcomes in NPs. Organizational support was identified as the most important of these factors. Organization administrators play an important role in developing strategies and policies for improving organizational support in support of NP practice. Favorable organizational support positively affects NP job satisfaction and decreases intention to change jobs, which improves NP retention rates and stabilizes NP manpower, providing positive outcomes for administrators, NPs, and patients.
